# Increased visceral fat area to skeletal muscle mass ratio is positively associated with the risk of metabolic dysfunction-associated steatotic liver disease in a Chinese population

**DOI:** 10.1186/s12944-024-02100-5

**Published:** 2024-04-14

**Authors:** Chenbing Liu, Nan Li, Di Sheng, Yahong Shao, Lihong Qiu, Chao Shen, Zhong Liu

**Affiliations:** Department of Health Management Center, The First Affiliated Hospital of Zhejiang University School of Medicine, No.79 Qingchun Road, Hangzhou, 310003 China

**Keywords:** Visceral fat area, Skeletal muscle mass, Metabolic dysfunction-associated steatotic liver disease

## Abstract

**Background:**

The diagnosis and comprehension of nonalcoholic fatty liver disease (NAFLD), recently redefined as metabolic dysfunction-associated steatotic liver disease (MASLD) are gaining a better understanding. In this study, we examined the association between visceral fat area and skeletal muscle mass ratio (VSR) and the prevalence of MASLD in a Chinese population.

**Methods:**

A cross-sectional study was conducted involving 10,916 individuals who underwent bioelectrical impedance analysis, along with anthropometric and biochemical measurements, from January 2022 to June 2023. According to the VSR distribution, sex-specific quartiles of VSR within the study population were defined. Linear trend tests were performed for the categorized VSR variables. Logistic regression models were performed to estimate the odds ratio and 95% confidence intervals between VSR distribution and MASLD prevalence stratified by sex.

**Results:**

The prevalence of MASLD was 37.94% in the overall population (56.34% male), and it gradually increased with higher VSR levels in both genders (*P* < 0.001). Logistic regression analysis demonstrated a significant association between VSR and MASLD prevalence after adjusting for confounders. The odds ratio (95% confidence interval) for MASLD, comparing the lowest to the highest VSR quartile, was 3.159 (2.671, 3.736) for men and 2.230 (1.764, 2.819) for women (all *P* < 0.001). Restricted cubic splines also indicated significant non-linear relationships between VSR and MASLD prevalence.

**Conclusions:**

VSR is positively associated with the prevalence of MASLD in this Chinese population, with a notably higher risk for men as VSR increases compared to women.

**Supplementary Information:**

The online version contains supplementary material available at 10.1186/s12944-024-02100-5.

## Introduction

Nonalcoholic fatty liver disease (NAFLD) stands as the most prevalent liver condition worldwide, with a prevalence rate around 38% in recent years [1, 2]. NAFLD is intricately linked to type 2 diabetes mellitus, obesity, metabolic syndrome, increasing the odds of liver-specific conditions [2–4]. NAFLD nomenclature has been under discussion recently. Following rounds of Delphi surveys, the new designation, metabolic dysfunction-associated steatotic liver disease (MASLD), has been selected to replace NAFLD [5]. Recently, two cohort studies found minimal disparity in the prevalence between MASLD and NAFLD, suggesting previous NAFLD research remain applicable with the new MASLD definition [6, 7]. Additionally, a large cohort study concluded that the change in nomenclature did not impact credibility of research findings presented under the NAFLD terminology in previous decades [8]. Therefore, the new term and criteria were utilized in the study while referencing some earlier NAFLD research.

Obesity, particularly abdominal obesity, is strongly related to NAFLD [9, 10]. The measurement of visceral fat area (VFA) provides a precise and consistent assessment of abdominal obesity [11, 12]. The presence of visceral fat has been associated with an increasing likelihood of developing NAFLD [13] and has become another contributing factor for metabolic syndrome and cardiovascular disease (CVD) [14]. Concurrently, reduced skeletal muscle mass (SMM), a key aspect of sarcopenia, also plays a role in increasing NAFLD risks [15].

Moreover, insulin resistance (IR) and NAFLD are closely linked [16]. Both visceral adipose tissue and SMM contribute to IR. SMM plays a significant role in the disposal of glucose mediated by insulin, and reduced SMM may induce relative IR [17, 18]. Similarly, visceral adipose tissue is closely linked with IR [19]. The combined effect of increased VFA and decreased SMM may exhibit a synergistic effect on metabolism, increasing the possibility of MASLD. Additionally, recent genetic insights, notably the identification of the TM6SF2 gene, contribute to our understanding of NAFLD development by revealing its role in hepatic lipid metabolism, particularly in the process of lipidating very low-density lipoprotein within a pre-Golgi compartment [20, 21]. To date, no research has explored the impact of both components in relation to MASLD in the Chinese population. Whether the combined effect of these aspects of body composition varies across sexes remains unclear.

The study seeks to evaluate the correlation between visceral fat area to skeletal muscle mass ratio (VSR) and the prevalence of MASLD within the Chinese population undergoing regular health examinations. The hypothesis posits that individuals with a higher VSR may have an increased prevalence of MASLD, given the potential synergistic effects of visceral adiposity and skeletal muscle alterations on metabolic health. In addition, the investigation aims to explore potential gender-specific differences in this association.

## Methods

### Study design and subjects

This cross-sectional study recruited participants who performed electrical impedance analysis (BIA) in the Health Management Center, the First Affiliated Hospital of Zhejiang University School of Medicine, between January 2022 and June 2023. A total of 13,815 subjects were included. The exclusion criteria included (1) incomplete anthropometric measurements or laboratory data (*n* = 185), or missing medical history data or personal health history data (*n* = 2664); (2) with a history of severe heart disease, brain disease, blood disease, lung disease, kidney disease, psychiatric disease, and malignancy (*n* = 50). In the end, 10,916 subjects met the inclusion criteria. The research obtained ethical approval from the First Affiliated Hospital of Zhejiang University School of Medicine. An informed consent waiver was granted by the ethics committee because anonymized retrospective data from health check-ups were utilized. The study adhered to the principles outlined in the Declaration of Helsinki.

### Clinical and lab measurements

Demographic data were gathered, and anthropometric measurements such as height, weight, and waist circumference were recorded. Body mass index (BMI) was calculated by dividing the weight (kg) by the square of the height (m). Body composition, including SMM and VFA, was assessed by the BIA method (InBody 770, Korea) [22]. To improve the accuracy of the result, subjects were required to fast for 12 h and refrain from drinking for 8 h before undergoing the measurement [23]. According to manufacturer’s instruction, subjects were light dressed and instructed to hold the analyzer’s handles, making contact with the electrodes on each limb. They extended their limbs to prevent their arms from touching the torso, kept their thighs from making contact and maintained a still position throughout the measurement [24]. The BIA method was validated as the reliable technique for assessing body composition, showing strong correlations with measurements obtained utilizing abdominal computed tomography and dual-energy X-ray absorptiometry (DXA), including VFA and SMM [25, 26]. In individuals with obesity, it proves sufficiently reliable for estimating fat mass, exhibiting a strong correlation and minimal bias compared to DXA [27]. Two cross-sectional studies, involving over 400,000 Korean adults, employed BIA as a dependable option for measuring skeletal muscle, further affirming its reliability in the diverse populations [28, 29]. In this study, VSR was computed as an indicator of MASLD risk by dividing the VFA (cm^2^) by SMM (kg).

Blood samples were collected following an 8-hour fasting period from subjects to measure the following parameters: aspartate aminotransferase (AST), alanine aminotransferase (ALT), γ-glutamyl transferase (GGT), low-density lipoprotein cholesterol (LDL-C), high-density lipoprotein cholesterol (HDL-C), total cholesterol (TC), triglyceride (TG), fasting plasma glucose (FPG), fasting insulin, glycated hemoglobin A1c (HbA1c), uric acid, homocysteine (hcy) and serum 25-hydroxyvitamin D (25(OH)D). All assessments were performed using standard laboratory methods. Insulin resistance index (HOMA-IR) was derived by multiplying FPG (mmol/L) by the fasting insulin level (µIU/mL), then dividing the result by 22.5 [30]. Central obesity was determined based on the criteria established by the World Health Organization: waist circumference (WC) of 94 cm or more for men and 80 cm or more for women [31].

### Diagnosis of metabolic dysfunction-associated steatotic liver disease

Hepatic steatosis was assessed using abdominal ultrasound performed by experienced radiologists. Steatosis diagnosis was established dichotomously based on the presence of a hyperechogenic liver parenchyma [32].

MASLD is defined as hepatic steatosis and one or more of the five cardiometabolic risk factors with no other causes of steatosis [5]:

i) body mass index ≥ 23 kg/m^2^ for Asians or WC > 94 cm for men and > 80 cm for women or ethnically adjusted;
ii) fasting serum glucose ≥ 5.6mmol/L(100 mg/dL) or 2-hour post-load glucose levels ≥ 7.8 mmol/L (≥ 140 mg/dL) or glycated hemoglobin ≥ 5.7% (39 mmol/L) or type 2 diabetes or treatment for type 2 diabetes;
iii) blood pressure ≥ 130/85 mmHg or specific anti-hypertensive drug treatment;
iv) plasma TG ≥ 1.70 mmol/L (150 mg/dL) or lipid-lowering treatment;
v) plasma HDL-C ≤ 1.0 mmol/L (40 mg/dL) for men and ≤ 1.3mmol/L (50 mg/dL) for women or lipid-lowering treatment.

### Statistical analysis

Categorical variables were indicated as the number of subjects (%), and chi-square test was preformed between groups. Normally distributed data are shown as means ± standard deviation values and comparison between groups was used by student’s t-test; otherwise indicated as inter-quartile range median (P25, P75) for non-normal distribution data and the Mann–Whitney U-test was conducted between groups. According to the VSR distribution, sex-specific quartiles of VSR within the study population were defined as follows: ≤2.26, 2.27 ~ 2.75, 2.76 ~ 3.29, and > 3.29 for men, and ≤ 2.85, 2.86 ~ 3.62, 3.63 ~ 4.88, > 4.88 for women. The first quartile representing the lowest VSR was used as the reference group. Based on logistic regression models, the odds ratio and 95% confidence intervals (95% CIs) were calculated to estimate the odds of MASLD associated with VSR distribution levels by sex. Model 1 was adjusted for age. Model 2 was adjusted for model 1 variables plus current smoking, current drinking and exercise (never versus occasional or regular). Model 3 was adjusted for model 2 variables plus TC, LDL-C, ALT, AST$,\gamma -$GGT, hcy, and uric acid. Linear trend analyses were conducted for the categorized VSR variables. Restricted cubic splines of continuous VSR with knots at 5th, 35th, 65th, and 95th percentiles were applied to graphically assess the potential non-linear association between VSR levels and MASLD prevalence following adjustment for all potential confounding factors. Moreover, subgroup analyses were performed to evaluate the impact of age, smoking, drinking, hcy and uric acid on MASLD outcomes among different gender groups. The potential interactions between each stratified variable and VSR levels were investigated using an interaction test in the subgroup analysis. All data analyses were conducted using SPSS 22.0 (SPSS Inc., Chicago, IL, USA) and R version 4.2.2, and *P* values < 0.05 were considered statistically significant.

## Results

### Baseline characteristics

The study comprised a total of 10,916 participants, including 6,150 males and 4,766 females. Table 1 displayed the baseline characteristics of study subjects categorized into MASLD and Non-MASLD groups. MASLD was prevalent in 37.94% of the participants. The findings revealed that the MASLD group had higher proportions of males, smokers, alcohol consumers, and individuals with a medical history of hypertension and diabetes compared to the Non-MASLD group (all *P* < 0.001). Additionally, in comparison to the Non-MASLD group, individuals in the MASLD group were older and exhibited higher for waist circumference, BMI, body fat percentage, VFA, VSR, TC, TG, systolic and diastolic blood pressure, ALT, AST, γ-GGT, LDL-C, hcy, FPG, HbA1c, HOMA-IR, and uric acid levels, while skeletal muscle index and HDL-C levels were lower (all *P* < 0.05). However, it should be noted that even if these values are higher in the MASLD group, a number of them still fall within the normal range.

**Table 1:** Comparison of subject baseline characteristics according to MASLD

	All subjects (*n* = 10,916)	Non-MASLD group (*n* = 6774)	MASLD group (*n* = 4142)	*P*
Age (years)	49.65±11.00	48.72±11.32	51.17±10.28	< 0.001
Male (%)	6150 (56.34)	3006 (44.38)	3144 (75.91)	< 0.001
Current smoking (%)	2296 (21.03)	1061 (15.66)	1235 (29.82)	< 0.001
Current drinking (%)	4142 (37.97)	1847 (29.59)	2295 (49.10)	< 0.001
Exercise (%)	2370(21.71)	1635(24.14)	735(17.35)	< 0.001
Hypertension history (%)	2028 (18.58)	814 (12.02)	1214 (29.31)	< 0.001
Diabetes history (%)	627 (5.74)	217 (3.20)	410 (9.90)	< 0.001
BMI (kg/m^2^)	23.96±3.17	23.04±2.85	25.45±3.10	< 0.001
WC (cm)	82.65±4.89	78.46±8.56	89.48±7.77	< 0.001
PBF (%)	27.99±5.75	27.51±5.88	28.77±5.45	< 0.001
VFA (cm^2^)	83.73±27.73	77.89±25.69	93.29±28.27	< 0.001
SMI (%)	39.67±3.61	39.74±3.73	39.56±3.40	< 0.05
VSR (cm^2^/kg)	3.03 (2.45, 3.94)	2.96 (2.38, 3.93)	3.10 (2.58, 3.95)	< 0.001
SBP (mmHg)	122.86±17.21	119.57±17.09	128.23±16.02	< 0.001
DBP (mmHg)	73.46±11.39	70.89±10.87	77.67±10.96	< 0.001
TC (mmol/L)	4.89±0.93	4.84±0.91	4.98±0.96	< 0.001
TG (mmol/L)	1.30 (0.91, 1.95)	1.06 (0.79, 1.48)	1.86 (1.35, 2.64)	< 0.001
HDL-C (mmol/L)	1.31±0.36	1.42±0.37	1.12±0.26	< 0.001
LDL-C (mmol/L)	2.74±0.74	2.71±0.73	2.79±0.77	< 0.001
Hcy (µmol/L)	9.50 (8.00, 11.30)	9.00 (7.50, 10.90)	10.20 (8.80, 11.90)	< 0.001
ALT (U/L)	18.00 (13.00, 27.00)	15.00 (12.00, 21.00)	25.00 (18.00, 37.00)	< 0.001
AST (U/L)	19.00 (16.00, 23.00)	18.00 (15.00, 21.00)	21.00 (17.00, 26.00)	< 0.001
$\gamma -$GGT (U/L)	22.00 (14.00, 38.00)	17.00 (12.00, 26.00)	34.00 (23.00, 56.00)	< 0.001
FPG (mmol/L)	4.79 (4.44, 5.21)	4.69 (4.37, 5.02)	5.02 (4.60, 5.61)	< 0.001
HbA1c (%)	5.60 (5.40, 5.90)	5.60 (5.30, 5.80)	5.80 (5.50, 6.20)	< 0.001
HOMA-IR	1.38 (0.95, 2.02)	1.12 (0.82, 1.22)	1.96 (1.43, 2.74)	< 0.001
SUA (µmol/L)	331.05±86.84	304.77±78.76	374.03±82.18	< 0.001
25(OH)D (nmol/L)	54.56±20.93	54.30±21.85	54.98±19.32	0.100

*Abbreviations* ALT, alanine aminotransferase; AST, aspartate aminotransferase; BMI, body mass index; DBP, diastolic blood pressure; FPG, fasting plasma glucose; $\gamma -$GGT:γ-glutamyl transferase; HbA1c, glycated hemoglobin; HDL-C, high-density lipoprotein cholesterol; Hcy, homocysteine; LDL-C, low-density lipoprotein cholesterol; MASLD: metabolic dysfunction-associated steatotic liver disease; PBF, percent body fat; SBP, systolic blood pressure; SMI, skeletal muscle mass index; SUA, serum uric acid; TC, total cholesterol; TG, triglyceride; VFA, visceral fat area; VSR, visceral fat area to skeletal muscle mass ratio; WC, waist circumference

### Prevalence of MASLD according to VSR values

**Fig. 1:**
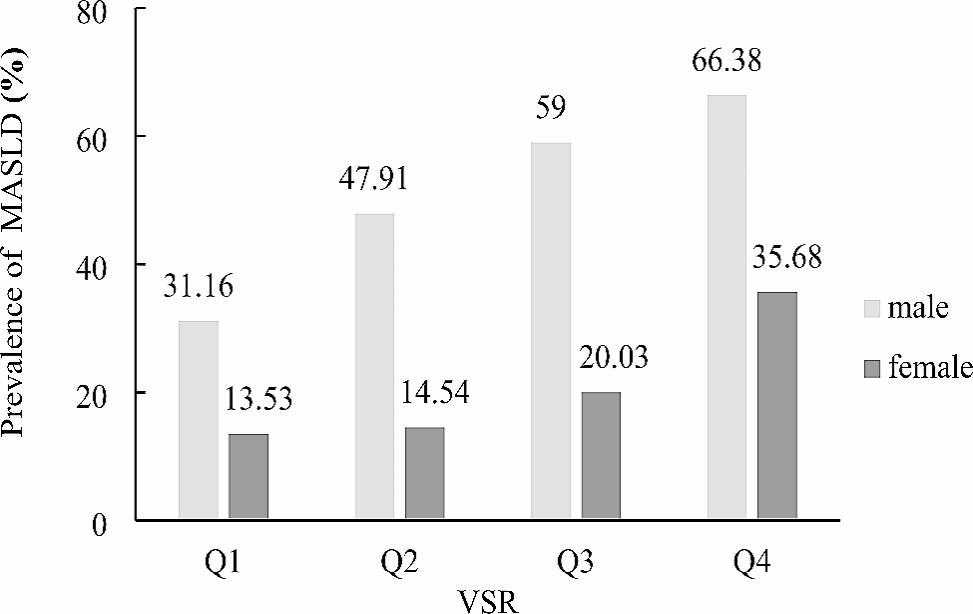
Prevalence of MASLD grouped by VSR quartiles in male and female. Quartiles of VSR in men, Q1: ≤ 2.26, Q2: 2.27 ~ 2.75, Q3: 2.76 ~ 3.29, Q4 > 3.29; quartiles of VSR in women, Q1: ≤2.85, Q2: 2.86 ~ 3.62, Q3: 3.63 ~ 4.88, Q4: >4.88. Abbreviations: MASLD, metabolic dysfunction-associated steatotic liver disease; VSR, visceral fat area to skeletal muscle mass ratio

Subjects were divided into quartiles according to their VSR values for different genders. Those in the highest VSR quartiles had lower SMM and higher VFA. In both genders, the prevalence of MASLD progressively increased with rising VSR levels (*P* < 0.001) (Fig. 1). In Table 2, following adjustment for confounding factors, the odds ratio (95% CIs) for MASLD, comparing the lowest to the highest VSR quartile, were 3.138 (2.652, 3.713) for males and 2.108 (1.664, 2.668) for females (all *P* < 0.001). Furthermore, the test for trend showed statistically significant results for both males and females (all *P* for trend < 0.001).

**Table 2:** OR with 95% CI for associations between VSR and MASLD according to sex

	VSR	N	Model 1	Model 2	Model 3
Male	Q1	1537	1.00 (ref.)	1.00 (ref.)	1.00 (ref.)
	Q2	1534	2.054 (1.772, 2.381) ***	2.037 (1.756, 2.362) ***	1.738 (1.478, 2.043) ***
	Q3	1544	3.229 (2.783, 3.747) ***	3.157 (2.718, 3.666) ***	2.570 (2.183, 3.026) ***
	Q4	1535	4.475 (3.842, 5.213) ***	4.333 (3.715, 5.053) ***	3.138 (2.652, 3.713) ***
*P* for trend			< 0.001	< 0.001	< 0.001
Female	Q1	1190	1.00 (ref.)	1.00 (ref.)	1.00 (ref.)
	Q2	1197	1.040 (0.820, 1.318)	1.021 (0.804, 1.297)	0.969 (0.765, 1.296)
	Q3	1188	1.472 (1.176, 1.844) **	1.436 (1.145, 1.800) **	1.295 (1.009, 1.662) *
	Q4	1291	2.760 (2.233, 3.411) ***	2.624 (2.120, 3.247) ***	2.108 (1.664, 2.668) ***
*P* for trend			< 0.001	< 0.001	< 0.001

*Note* Model 1: adjusted for age. Model 2: model 1 plus current smoking, drinking, and exercise. Model 3: model 2 plus total cholesterol, low-density lipoprotein cholesterol, homocysteine, uric acid, alanine aminotransferase, aspartate aminotransferase, and γ-glutamyl transferase. Quartiles of VSR in men, Q1: ≤ 2.26, Q2: 2.27 ~ 2.75, Q3: 2.76 ~ 3.29, Q4 > 3.29; quartiles of VSR in women, Q1: ≤2.85, Q2: 2.86 ~ 3.62, Q3: 3.63 ~ 4.88, Q4: >4.88. Compare to Q1, *P < 0.05, **P < 0.01, ***P < 0.001

**Fig. 2:**
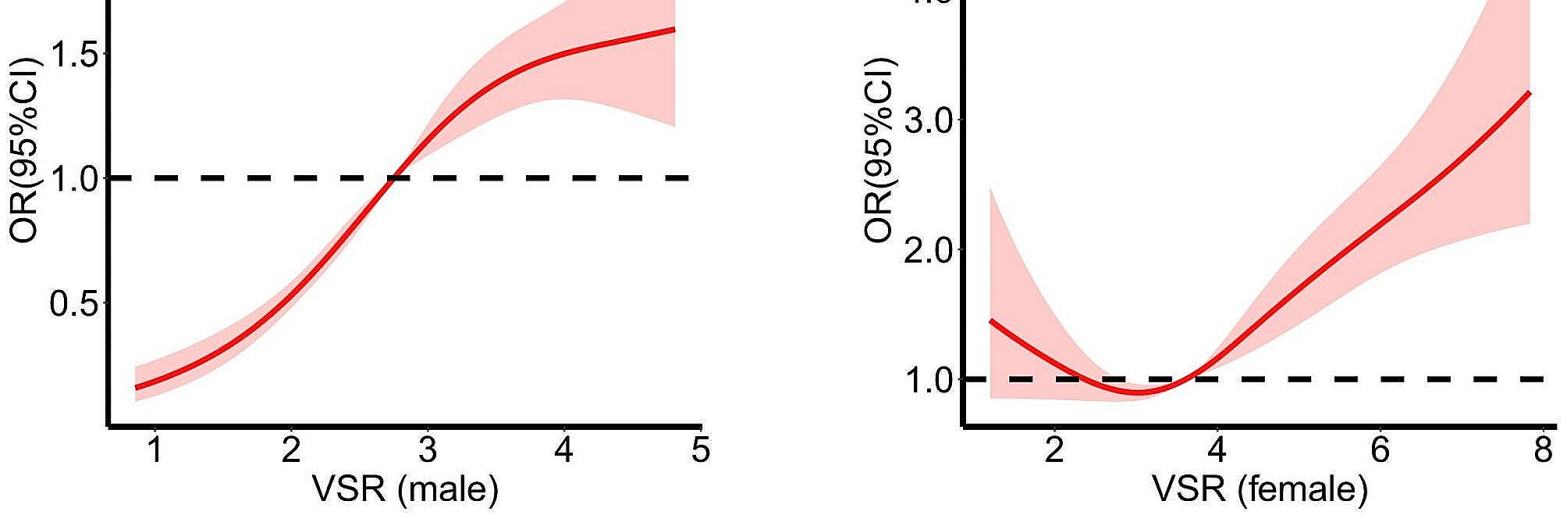
Non-linear relationships between VSR and prevalence of MASLD. (**A**) in male. (**B**) in female. Data were OR (solid line) and 95%CI (shadow area) from logistic regression analysis with restricted cubic splines. Abbreviations: CI, confidence intervals; MASLD, metabolic dysfunction-associated steatotic liver disease; OR, odds ratio; VSR, visceral fat area to skeletal muscle mass

Based on logistic regression results adjusted for confounders, a restricted cubic spline model with four knots was employed to assess the dose-response relationship between VSR and the prevalence of MASLD (Fig. 2). In males, the possibility of MASLD increased across the range of VSR. However, in females, the non-linear relationship between VSR and odds ratio showed a J-shaped pattern, with an overall tendency suggesting a positive association between VSR and MASLD prevalence (Fig. 2).

### Subgroup analysis

In the subgroup analysis, stratified analysis was conducted on the correlation between VSR and prevalence of MASLD in different genders (as shown in Table 3). The findings revealed a significant interaction: age and central obesity in both sex, uric acid in females, interact significantly with VSR (*P* for interaction < 0.05), increasing the prevalence of MASLD. However, due to the limited number of smokers, alcohol drinkers and individuals with high hcy levels, subgroup analysis was not conducted for these three factors in females.

**Table 3:** Subgroup analysis for the association of VSR with the prevalence of MASLD according to sex

	VSR	Q1 OR (95%CI)	Q2 OR (95%CI)	Q3 OR (95%CI)	Q4 OR (95%CI)	*P* for trend	*P *for interaction
**Male**	**Age**						0.011
	18 ~ 44 yr	1.00 (ref.)	2.152 (1.606, 2.884) ***	3.145 (2.327, 4.252) ***	3.753 (2.717, 5.186) ***	< 0.001	
	45 ~ 59 yr	1.00 (ref.)	1.674 (1.344, 2.085) ***	2.531 (2.027, 3.159) ***	2.932 (2.333, 3.686) ***	< 0.001	
	≥ 60 yr	1.00 (ref.)	1.313 (0.838, 2.056)	2.115 (1.371, 3.262) **	3.161 (2.074, 4.819) ***	< 0.001	
	**Current smoking**						0.586
	No	1.00 (ref.)	1.649 (1.344, 2.023) ***	2.817 (2.293, 3.461) ***	3.082 (2.493, 3.811) ***	< 0.001	
	Yes	1.00 (ref.)	1.920 (1.468, 2.510) ***	2.193 (1.674, 2.873) ***	3.285 (2.484, 4.345) ***	< 0.001	
	**Current drinking**						0.448
	No	1.00 (ref.)	1.702 (1.412, 2.051) ***	2.839 (2.349, 3.433) ***	3.108 (2.559, 3.776) ***	< 0.001	
	Yes	1.00 (ref.)	1.867 (1.339, 2.602) ***	1.938 (1.403, 2.677) ***	3.145 (2.235, 4.426) ***	< 0.001	
	**Exercise**						0.548
	No	1.00 (ref.)	1.784 (1.472, 2.163) ***	2.612 (2.156, 3.164) ***	3.366 (2.771, 4.089) ***	< 0.001	
	Yes	1.00 (ref.)	1.617 (1.192, 2.192) **	2.534 (1.844, 3.481) ***	2.426 (1.710, 3.441) ***	< 0.001	
	**Central obesity**						< 0.001
	No	1.00 (ref.)	1.722 (1.452, 2.042) ***	2.398 (2.011, 2.859) ***	2.436 (2.021, 2.936) ***	< 0.001	
	Yes	1.00 (ref.)	1.153 (0.588, 2.261)	1.186 (0.645, 2.183)	1.864 (1.020, 3.049) *	< 0.001	
	**Hyperhomocysteinemia**						0.848
	No	1.00 (ref.)	1.722 (1.455, 2.037) ***	2.515 (2.120, 2.983) ***	3.073 (2.575, 3.667) ***	< 0.001	
	Yes	1.00 (ref.)	2.113 (1.148, 3.890) *	3.311 (1.869, 5.864) ***	4.100 (2.309, 7.283) ***	< 0.001	
	**Hyperuricemia**						0.067
	No	1.00 (ref.)	1.916 (1.592, 2.307) ***	2.852 (2.364, 3.441) ***	3.389 (2.792, 4.114) ***	< 0.001	
	Yes	1.00 (ref.)	1.308 (0.941, 1.820)	1.896 (1.364, 2.635) ***	2.717 (1.932, 3.821) ***	< 0.001	
**Female**	**Age**						< 0.001
	18 ~ 44 yr	1.00 (ref.)	1.335 (0.710, 2.510)	1.474 (0.801, 2.709)	3.760 (2.103, 6.720) ***	< 0.001	
	45 ~ 59 yr	1.00 (ref.)	0.981 (0.679, 1.417)	1.520 (1.083, 2.132) *	2.596 (1.871, 3.602) ***	< 0.001	
	≥ 60 yr	1.00 (ref.)	0.668 (0.403, 1.108)	0.786 (0.476, 1.299)	0.967 (0.625, 1.495)	0.117	
	**Exercise**						0.168
	No	1.00 (ref.)	0.865 (0.651, 1.148)	1.147 (0.879, 1.496)	1.927 (1.502, 2.472) ***	< 0.001	
	Yes	1.00 (ref.)	2.322 (1.044, 5.163) *	3.597 (1.665, 7.772) **	4.323 (1.985, 9.419) ***	< 0.001	
	**Central obesity**						< 0.001
	No	1.00 (ref.)	1.114 (0.765, 1.621)	1.365 (0.948, 1.966)	1.928 (1.321, 2.861) **	0.002	
	Yes	1.00 (ref.)	0.853 (0.549, 1.323)	0.830 (0.562, 1.226)	1.032 (0.726, 1.465)	0.048	
	**Hyperuricemia**						0.023
	No	1.00 (ref.)	1.059 (0.816, 1.373)	1.369 (1.707, 1.752) *	2.441 (1.936, 3.078) ***	< 0.001	
	Yes	1.00 (ref.)	0.773 (0.139, 4.306)	0.763 (0.146, 3.984)	1.392 (0.299, 6.488)	0.216	

*Note* *P < 0.05, **P < 0.01, ***P < 0.001

## Discussion

This cross-sectional study examined the relationship between VSR and the prevalence of MASLD in Chinese adults underwent health check-up. We found decreased SMM and increased VFA may have a synergistic effect on the prevalence of MASLD. As VSR elevated from Q1 to Q4, there was a significant rise in MASLD prevalence even after adjusting for confounders. Additionally, the relation between VSR and MASLD was more pronounced in men than women. To our knowledge, this research represents the first investigation into the association of VFA and SMM in relation to the prevalence of MASLD, a new term chosen to replace NAFLD, across both genders in a Chinese population. Several cohort studies have shown that earlier research into NAFLD remain applicable even with the adoption of the new MASLD definition [6–8]. The results align with prior research indicating that visceral fat and skeletal muscle may exert opposing influences on each other and both significantly contribute to the risk of NAFLD [15, 33–35], a pattern observed consistently across different populations including the Chinese. Specifically, a study conducted among Chinese patients with type 2 diabetes observed strong associations between low SMM and high visceral adiposity with the highly prevalent lean NAFLD [36]. Another study conducted among middle-aged and elderly Chinese adults identified the fat-to-muscle ratio as an independent contributor to the risk of NAFLD [37]. Additionally, Yu et al.’s study concluded that a lower appendicular skeletal muscle mass to visceral fat area ratio is linked to a higher prevalence of subtypes of metabolic associated fatty liver disease [38]. Some mechanisms could elucidate the combined impact of reduced SMM and increased VFA on NAFLD risk, including IR, impaired lipid metabolism, and low-grade inflammation [24, 39]. Skeletal muscles serve as a site for insulin-mediated glucose uptake. Therefore, the decrease in skeletal muscle mass can result in reduced insulin sensitivity, a key factor in the development of steatotic liver disease [17]. In addition, decreased muscle mass hinders the effective clearance of TG and free fatty acids, resulting in elevated lipid transport to the liver and subsequent hepatic fat accumulation. Steatotic liver disease often involves low-grade inflammation, where certain inflammatory mediators originating from the liver and other pro-inflammatory cytokines have been associated with decreased muscle mass [40]. Visceral fat, on the other hand, disrupts adipocyte function and adipocytokine secretion, potentially increasing proinflammatory cytokines like tumor necrosis factor-alpha and interleukin-6, which are correlated with muscle atrophy and could heighten the risk of progressing NAFLD [41]. Therefore, as visceral adipose tissue increases and SMM decreases, the ability of insulin to mediate glucose uptake and storage in both skeletal muscle and adipose tissue decreases. This phenomenon may contribute to the development of NAFLD.

VSR, combining two body composition measures can be used as a proxy for sarcopenic obesity. The current study found that a higher VSR value was in relation to a greater prevalence of MASLD compared to the lower VSR value group, irrespective of gender. However, our research found gender disparities, with the association between VSR and MASLD being stronger in men compared to women. The J-shaped association was observed only in women, indicating when VSR was around 2.2–3.7, the association of VSR and MASLD was relatively weak in women, but the risk began to rise significantly afterward. This contrasts with previous research where associations are typically stronger in women, leading to increased VSR levels, and are more likely to develop sarcopenic obesity, cardiometabolic diseases, and NAFLD [39, 42, 43]. The mechanisms are mainly explained by the variations in fat distribution and skeletal muscle mass, which are influenced by varying levels of sex hormones between different genders [44]. Men are predisposed to central obesity as a result of increased visceral fat accumulation, while women tend to exhibit a ‘pear-shaped’ body profile, characterized by preferential fat deposition in the lower body [45]. When VSR falls within the first and second quartiles, women are more likely to maintain normal levels of SMM and VFA. Furthermore, there were fewer women than men (*n* = 4766 versus *n* = 6150), and the rate of MASLD prevalence is a much higher among men than among women (75.91% versus 24.09%). Given the variations in diagnostic methods for hepatic steatosis and sarcopenia, a conclusion has yet to be reached. Further investigation utilizing comprehensive assessments of fat distribution and SMM measurements will provide insights into the varying impact of VSR on MASLD prevalence between genders.

The diagnostic criteria of MASLD requires one or more of the five cardiometabolic risk factors, including weight status, glucose/HbA1c level, blood pressure, HDL-C, and TG levels. These risk factors can be influenced by both SMM and VFA with different pathways. SMM is linked to improved glucose metabolism and insulin sensitivity, reducing risk of abnormal glucose/HbA1c levels. Furthermore, it contributes to overall energy expenditure, facilitating weight management and favorable lipid profiles. In contrast, VFA primarily impacts cardiometabolic risk factors through mechanisms related to inflammation, IR, and unfavorable lipid metabolism. The release of pro-inflammatory substances, IR, and adverse lipid profiles are consequences of excessive visceral fat, resulting in an elevated risk of type 2 diabetes mellitus and CVD. These two components of body composition have unique roles in regulating cardiometabolic health. When examining gender disparities, it’s evident that men tend to accumulate more abdominal fat [45], a known predictor of unfavorable metabolic characteristics that elevate the risk of CVD. Conversely, increased fat deposition in the legs may be linked to a reduced risk of metabolic dysfunction [46–48]. These observations highlight the significance of considering fat distribution patterns in the onset of cardiometabolic diseases, potentially explaining the higher prevalence of MASLD among men.

In subgroup analysis, it was observed that only uric acid in women interacted with VSR, increasing the possibility of MASLD. Yang et al. found that uric acid levels were positively related to NAFLD prevalence, with a stronger correlation observed in elderly women relative to men [49]. Similarly, in a large cross-sectional study, women exhibited a more robust correlation between uric acid levels and elevated ALT compared to men [50]. Conversely, Lin’s group found that hyperuricemia was associated with increased ALT levels in young men, but this association was not observed in women [51]. Although the mechanism underlying this sex difference requires further investigation, it suggests that individuals with elevated uric acid levels might warrant screening for MASLD prevalence.

### Study strengths and limitations

The study has several strengths. Firstly, this is the first study to explore the combined impact of VFA and SMM on MASLD, a new term chosen to replace NAFLD, among a large number of Chinese population. Second, this study illuminates gender disparities in these associations, taking into account the substantial differences in body composition between men and women. Third, restricted cubic spline curves are used to vividly illustrate the non-linear associations between VSR and the prevalence of MASLD as they are not linearly related. These findings underscore the relevance of considering both visceral fat and SMM in assessing MASLD prevalence, pointing towards the need for a more gender-specific approach in disease prevention and intervention. Dietitians and other healthcare professionals can utilize these findings to optimize personalized dietary interventions and nutrition prescriptions, ultimately advancing the field’s knowledge and enhancing strategies for the prevention and management of MASLD.

This study also has several limitations. First of all, while our findings suggest an association between VSR and MASLD prevalence, the study design prevents us from drawing definitive conclusions regarding causality. Further longitudinal studies are necessary to evaluate how VSR contributes to the prevalence of MASLD and to provide a more comprehensive understanding of their relationships. Second, BIA results may be influenced by various factors, including fluid status, malnutrition, and pregnancy [52]. Nonetheless, any inaccuracies in the BIA assessment would apply uniformly to all study participants. In addition, while BIA may not be the gold standard for assessing muscle mass, it offers simple, cost-effective, and dependable estimates of SMM [53]. When conducting the measurements, the hydration level and food intake were regulated to control the variables. Finally, the study population consist of only healthy Chinese ethnicity, who typically have a great access to healthcare facilities and are more likely to be motivated to enhance their health for various reasons. However, it’s important to note that this population may not fully reflect the broader demographic variations. Future comparative research involving diverse ethnic populations is needed to confirm the findings, enhance the results’ generalizability and gain a better understand the nuances of the outcomes observed.

## Conclusions

In summary, the current study reveals a notable positive relationship between high VSR and MASLD prevalence, particularly notable in males compared to females. This association remains independent of age, lipid levels, and other potential confounding factors within a large Chinese ethnic population. These findings underscore the clinical relevance of considering VSR as a potential risk factor for MASLD. Healthcare providers can tailor interventions to target both visceral fat reduction and skeletal muscle preservation or enhancement, potentially through lifestyle modifications, exercise programs, and dietary interventions. Further research involving diverse ethnic groups is necessary to validate and replicate the findings.

### Electronic supplementary material

Below is the link to the electronic supplementary material.

Supplementary Material 1

## Data Availability

No datasets were generated or analysed during the current study.

## Abbreviations

ALT: Alanine aminotransferase
AST: Aspartate aminotransferase
BIA: Bioelectrical impedance analysis
BMI: Body mass index
CVD: Cardiovascular disease
DBP: Diastolic blood pressure
DXA: Dual-energy X-ray absorptiometry
FPG: Fasting plasma glucose
γ-GGT: γ-glutamyl transferase
HbA1c: Glycated hemoglobin
Hcy: Homocysteine
HDL-C: High-density lipoprotein cholesterol
HOMA-IR: Insulin resistance index
IR: Insulin resistance
LDL-C: Low-density lipoprotein cholesterol
MASLD: Metabolic dysfunction-associated steatotic liver disease
NAFLD: Nonalcoholic fatty liver disease
PBF: Percent body fat
SBP: Systolic blood pressure
SMI: Skeletal muscle mass index
SMM: Skeletal muscle mass
SUA: Serum uric acid
TC: Total cholesterol
TG: Triglyceride
VFA: Visceral fat area
VSR: Visceral fat area to skeletal muscle mass ratio
WC: Waist circumference
