# Supplementary material for: Increased visceral fat area to skeletal muscle mass ratio is positively associated with the risk of metabolic dysfunction-associated steatotic liver disease in a Chinese population
Source: Lipids Health Dis. 2024 Apr 14;23:104. doi: 10.1186/s12944-024-02100-5 (PMC11016208; doi:10.1186/s12944-024-02100-5)
Supplement: Supplementary file 1 — Supplementary Material 1 [file 12944_2024_2100_MOESM1_ESM.pdf]

Increased visceral fat area to skeletal muscle mass ratio is positively associated with the risk of metabolic dysfunction-associated steatotic liver disease in a Chinese population

ORIGINALITY REPORT

8%
SIMILARITY INDEX

PRIMARY SOURCES

	Source	Words	%
1	eprints.soton.ac.uk<br>Internet	48 words —	2%
2	Ji Yeon Seo, Eun Ju Cho, Min Joo Kim, Min-Sun Kwak, Jong In Yang, Su Jin Chung, Jeong Yoon Yim, Ji Won Yoon, Goh Eun Chung. "The relationship between metabolic dysfunction-associated fatty liver disease and low muscle mass in an asymptomatic Korean population", Journal of Cachexia, Sarcopenia and Muscle, 2022<br>Crossref	35 words —	1%
3	Handbook of Anthropometry, 2012.<br>Crossref	20 words —	1%
4	Metabolic Syndrome, 2016.<br>Crossref	19 words —	1%
5	lipidworld.biomedcentral.com<br>Internet	19 words —	1%
6	pubag.nal.usda.gov<br>Internet	18 words —	1%
7	bmjopen.bmj.com<br>Internet	16 words —	1%

8 buscador.una.edu.ni
Internet
15 words — 1%

9 Xiaojie Cai, Menghui Liu, Xingfeng Xu, Shaozhao Zhang et al. "Cardiovascular effects of weight loss in old adults with overweight/obesity according to change in skeletal muscle mass", Journal of Cachexia, Sarcopenia and Muscle, 2023
Crossref
14 words — 1%

10 Zhibin Li, Mingzhu Lin, Changqin Liu, Zheng Chen, Dongmei Wang, Xiulin Shi, Shuyu Yang, Xuejun Li. " The rs4686434 variant in the locus is associated with intrahepatic triglyceride content in obese Chinese adults ", Journal of Diabetes, 2018
Crossref
14 words — 1%

EXCLUDE QUOTES	OFF	EXCLUDE SOURCES	< 1%
EXCLUDE BIBLIOGRAPHY	ON	EXCLUDE MATCHES	< 6 WORDS
